# Phosphorus-mineralizing Communities Reflect Nutrient-Rich Characteristics in Japanese Arable Andisols

**DOI:** 10.1264/jsme2.ME18043

**Published:** 2018-09-29

**Authors:** Kazumori Mise, Kazuki Fujita, Takashi Kunito, Keishi Senoo, Shigeto Otsuka

**Affiliations:** 1 Department of Applied Biological Chemistry, Graduate School of Agricultural and Life Sciences, The University of Tokyo 1–1–1 Yayoi, Bunkyo-ku, Tokyo 113–8657 Japan; 2 Department of Biological Sciences, Graduate School of Science, The University of Tokyo 2–11–16 Yayoi, Bunkyo-ku, Tokyo 113–0032 Japan; 3 Department of Environmental Sciences, Faculty of Science, Shinshu University 3–1–1 Asahi, Matsumoto 390–8621 Japan; 4 Collaborative Research Institute for Innovative Microbiology, The University of Tokyo 1–1–1 Yayoi, Bunkyo-ku, Tokyo 113–8657 Japan

**Keywords:** soil, microbial community structure, phosphorus, phosphatase

## Abstract

Elucidating the soil phosphorus cycle driven by soil microbes is a vital question in soil microbial ecology. The Japanese arable Andisols, occupying half of the Japanese cropland, are known for their high phosphorus sorption capacity. However, limited information is currently available on microbially driven phosphorus mineralization in arable Andisols. We herein report that the phosphorus-mineralizing community in the Japanese arable Andisols showed characteristic distribution and composition patterns, from those in other types of soils. We performed a chemical analysis and microbial community analysis of 43 arable Andisols along the Japanese archipelago. Soil phosphomonoesterase activities measured at pH 11 were approximately 70% of those at pH 6.5, which indicates that alkaline phosphatase contributes to phosphorus cycling, although most soil samples were acidic. Functional gene predictions based on 16S rRNA gene sequencing indicated that the alkaline phosphatase gene *phoD* was more abundant than other alkaline phosphatase genes and, thus, plays major roles. Hence, amplicon sequencing targeting *phoD* was performed and the results obtained showed that alphaproteobacterial *phoD* was dominant. This is in contrast to previously reported *phoD* compositions in other soils and may be attributed to the nutrient conditions in arable Andisols, which favor copiotrophic *Alphaproteobacteria*. Furthermore, the composition of *phoD* correlated with soil pH and bioavailable phosphorus concentrations rather than carbon or nitrogen concentrations. These results were partly different from previous findings, varying in the soil types and geographic ranges of sampling sites. Collectively, the present results indicate that the phosphorus-mineralizing community in the Japanese arable Andisols is regulated differently from those in other soil types.

Microbes play vital roles in nutrient cycling, and elucidating their contribution to phosphorus turnover in soil is a fundamental question in soil ecology and microbiology. In some environments, such as tropical rain forest soil and acidic soil, phosphorus has been identified as the limiting factor for soil microbes and plants (9, 29). Additionally, more soil environments have become phosphorus-limited rather than nitrogen-limited because the atmospheric nitrogen supply to soil, which originates from human activity, has become larger than the phosphorus supply (53). Hence, phosphorus cycling is a key factor in soil microbial activity and its functions. Therefore, the elucidation of phosphorus cycling is essential for understanding, predicting, and controlling the dynamics of soil ecosystems.

Soil phosphorus may be classified into one of the following three forms: free phosphate ions in soil solution (generally scarce in soil), inorganic phosphorus (for example, adsorbed on Fe and Al oxides, or insoluble metallophosphates, such as FePO_4_ and Ca_3_[PO_4_]_2_) and organic phosphorus (*e.g.*, phosphomonoesters, phosphodiesters, and phytate) (47). Organic phosphorus is primarily mineralized by microbial extracellular phosphomonoesterase (37), which has been shown to enhance phosphorus availability in soil (40). In prokaryotes, the production and secretion of phosphomonoesterase is regulated by the expression of the Pho regulon, which is enhanced in response to a scarcity of phosphorus. Thus, phosphomonoesterase genes within the Pho regulon have been intensively investigated using molecular ecology techniques (3, 8, 20, 22, 43, 46, 50, 54). More specifically, the community composition of prokaryotes harboring *phoD*, one clade of extracellular alkaline phosphatase genes, was found to be affected by multiple environmental factors, including soil pH (43), land usage (44), and phosphorus fertilization (50). However, these *phoD*-targeting studies are based on the assumption that *phoD* is the predominant clade of extracellular phosphatase, an inference from a comparative genomic analysis (50). Additionally, the product of the *phoD* gene is alkaline phosphatase with an optimum pH in the alkaline range (2), which suggests that *phoD* does not represent microbially-mediated phosphorus mineralization in acidic or neutral soil.

A high proportion of phosphate fertilizer amendments are immobilized in agricultural Andisols with a high phosphorus sorption capacity, which are distributed along the circum-Pacific volcanic belt (10), and, thus, only a small part of amended phosphorus is taken up by crop plants (35). Thus, large amounts of fertilizer amendments are needed in order to increase crop productivity. Recent studies demonstrated that abundant amounts of phosphorus accumulate in highly-fertilized agricultural Andisols (11, 39). In spite of the above-described characteristics of phosphorus cycling in arable Andisols, limited information is currently available on the microbial community driving phosphorus cycling in arable Andisols.

The purpose of the present study was to investigate the microbial community driving phosphorus mineralization in arable Andisols. More specifically, we aimed to address the following three questions: (i) whether alkaline phosphatase plays a major role in mineralizing organic phosphorus in arable Andisols, which are generally acidic or circumneutral; (ii) what type of prokaryote drives phosphorus mineralization in arable Andisols; (iii) what kind of environmental factor affects the phosphorus-mineralizing prokaryotic community. In order to achieve this, we conducted a biochemical analysis and microbial community analysis of forty-three arable Andisols collected along the Japanese archipelago. We compared soil phosphomonoesterase activities under weak-acidic and alkaline conditions in order to estimate the activity of alkaline phosphatase in low pH (question [i]). Based on the results obtained, we conducted functional gene prediction, which suggested that the *phoD*-targeting approach was effective for our samples. Therefore, we performed the amplicon sequencing of *phoD*, and analyzed the relationship between the composition of *phoD* and soil environmental factors (questions [ii],[iii]).

## Materials and Methods

### Sample collection

**Fig. 1:**
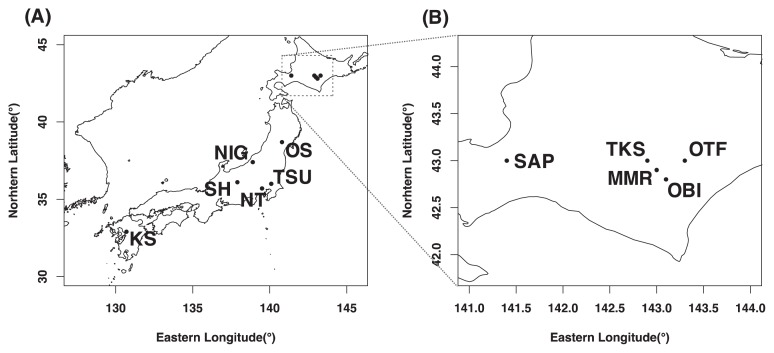
Locations of soil sampling sites. Alphabetic codes indicate site names (see Table S1). Map (A) indicates all the sites and (B) shows a magnification of the area surrounded by the dotted square.

Arable soil samples (Andisols or Kuroboku soils in the Japanese soil taxonomy system) were collected from the Ap horizon in 43 agricultural fields located in eleven regions in Japan between June to August, 2016. These sampling sites were distributed across the Japan archipelago: Tokachi-shimizu (abbreviated hereafter as TKS), Memuro (MMR), Otofuke (OTF), Obihiro (OBI), Sapporo (SAP), Osaki (OS), Niigata (NIG), Tsukuba (TSU), Nishi-Tokyo (NT), Shiojiri (SH), and Koshi (KS). Detailed sampling sites and land usage conditions are shown in Fig. 1 and Table S1. Collected samples were chilled at 4°C, packed in coolers, sent to the laboratory, and sieved through a 2-mm mesh for thorough homogenization. Some of the sieved samples were air-dried for soil chemical analyses, and the rest was stored at −30°C for enzyme activity measurements and microbial community analyses.

### Soil chemical analyses

Soil pH was measured using a glass electrode in a 1:2.5 (w/v) soil-water suspension. Soil moisture was estimated gravimetrically by drying the soil sample at 105°C for 10 h. Total carbon and nitrogen concentrations were measured by dry combustion using a Nitrogen and Carbon analyzer (Thermo Finnigan Flash EA1112; Thermo Fisher Scientific, Waltghram, MA, USA).

Phosphorus was extracted according to Truog’s method (51), which has been the most frequently employed extraction method among Japanese agricultural practical studies (48). Additionally, phosphorus concentrations measured using this method have been reported to reflect the microbial phosphorus demand (21). Briefly, soil samples were suspended in 0.001 M of H_2_SO_4_ solution (pH adjusted to 3.0 using [NH_4_]_2_SO_4_ solution) and then shaken for 30 min. This extraction solvent was designed to have approximately the same extraction strength as plant root exudates (51). The extracts were filtered and phosphorus concentrations were subsequently measured using the molybdate blue method (36).

### Soil enzyme activity measurements

Soil alkaline and acid phosphatase (phosphomonoesterase) activities were measured in order to evaluate the phosphorus demand of the microbial community. Since phosphomonoesterase is essential in mineralizing not only phosphomonoester, but also phosphodiester (49), its activity represents the mineralization process of soil phosphorus. Phosphomonoesterase activity was measured in modified universal buffer (MUB) of pH 6.5 (acid phosphatase) or 11 (alkaline phosphatase) using *p*-nitrophenylphosphate as the substrate after a 1-h incubation at 37°C (49). In order to evaluate total microbial activity, we measured β-D-glucosidase activity as an alternative to measuring soil microbial abundance based on the 16S rRNA gene copy number because nucleotide extraction efficiency from Andisols is typically very low (23) and strongly dependent on the soil type (56) such that the gene copy number in extracted soil DNA may not accurately represent microbial abundance. β-D-glucosidase activity was measured in MUB of pH 6.0 using *p*-nitrophenyl-β-D-glucopyranoside as the substrate (49). All enzyme measurements were performed using frozen and thawed soil. Of note, freeze-thawing does not have major impact on soil enzyme activities (32, 52).

### DNA extraction and 16S rRNA gene amplicon sequencing

Total DNA was extracted from approximately 500 mg of soil using the FastDNA SPIN Kit for Soil (Qbiogene, Carlsbad, CA, USA). We followed the standard protocol recommended by the manufacturer; however, 0.5 μL of 2% (w/v) casein solution (dissolved in 300 mM sodium phosphate, pH adjusted to 8.0, autoclaved) mg^−1^ of soil was added before cell lysis in order to inhibit DNA absorption by soil allophanic structures (45). Extracted DNA was electrophoresed on a 1.5% agarose gel for further purification, and DNA bands were excised from the gel and then subjected to the Wizard^®^ SV Gel and PCR Clean-Up System (Promega, Madison, WI, USA).

The preparation of extracted DNA for 16S rRNA gene sequencing was performed following the protocol described by Caporaso *et al.* (7), with modifications to PCR conditions (Table S2). In brief, the V4 region of the 16S rRNA gene was amplified with the primers 515f and 806r (6). These primers included the adaptor and index sequences required for Illumina sequencing-by-synthesis. The resulting barcoded PCR products were mixed in approximately equimolar amounts and sequenced on Illumina MiSeq using Reagent Kit V2 (500 cycles; Illumina, San Diego, CA, USA).

### Bioinformatics analyses of 16S rRNA gene amplicons

Illumina sequencing provided 8,441,310 reads. Low quality regions were trimmed, followed by the merging of paired–end reads and quality filtering (passing only sequences with expected errors of 0.5 bases or less) using USEARCH ver 9.2.64 (13). Filtered sequences were chimera-checked with the UCHIME2 algorithm (15), using Greengenes 13_8 (34) as the reference database. Chimera-checked sequences were subjected to operational taxonomic unit (OTU) clustering, at a similarity threshold of 97% or more (7), using the UPARSE-OTU algorithm (14) implemented in USEARCH ver 9.2.64. Taxonomic annotation was attached to the representative sequence of each OTU (*i.e.*, the most frequently observed nucleotide sequence within the OTU) using the RDP Classifier implemented in MacQIIME ver 1.9.1 (5), with a confidence threshold of 0.8; Greengenes 13_8 97% OTU representative sequences were employed as the reference database. OTUs annotated either as “f__mitochondria”, “c__Chloroplast”, “unassignable”, or “k__” (*i.e.*, unidentifiable at the kingdom or domain level) were excluded from further analyses. The representative sequences of all OTUs were aligned using the PyNAST algorithm (4), followed by the construction of a phylogenetic tree based on the FastTree algorithm (41) in preparation for down-stream statistical analyses.

Based on the 16S rRNA gene composition, functional gene content was independently predicted using two different tools based on different algorithms, namely, PICRUSt (30) and Piphillin (27). The numbers of functional genes were estimated using KEGG orthology (KO). In further analyses, we focused on KOs associated with three clades of alkaline phosphatase genes, namely, *phoA* (K01077), *phoD* (K01113), and *phoX* (K07093).

### Amplicon sequencing targeting the alkaline phosphatase gene

Metagenomic predictions indicated that *phoD* was significantly more abundant than *phoA* and *phoX* (Fig. 3). Hence, in order to characterize microbially driven phosphorus mineralization in each soil, the diversity of *phoD* was adopted as a marker and investigated.

The sequencing of *phoD* amplicons was conducted as follows. PCR was performed with PHOD-F733/R1083 primers, which are estimated to be less biased than other primer sets published to date (43). The size and quality of the amplicons were checked using electrophoresis, followed by the ligation of adaptor and barcode sequences with the NEB UltraDNA Library Prep Kit for Illumina (New England Biolabs, Ipswich, MA, USA). All amplicons were mixed and sequenced on Illumina MiSeq using Reagent Kit V2 (500 cycles; Illumina).

### Bioinformatics analyses of *phoD* amplicons

Illumina sequencing provided 11,906,472 reads. The merging of paired-end reads and the quality control procedures used were the same as those for the 16S rRNA gene amplicons. Reads that had stop codons or did not match the primer sequences were eliminated. The sequences obtained were subjected to OTU clustering at a similarity threshold of 75% identity or more (50) using the UCLUST algorithm implemented in USEARCH ver 9.2.64. The representative sequences of the OTUs (DOI: 10.6084/m9.figshare.6267686) were translated into amino acid sequences and subjected to a blastp search against the NCBI non-redundant protein (nr) database (as of July 1, 2017) with a maximum E-value of 1E-05 and minimum bit score of 100.

The blastp search annotated some OTUs as proteins other than phosphatase, including hypothetical proteins, making it difficult to analyze the phylogeny of *phoD* (blastp results are shown in Table S3). In order to overcome this issue, we constructed a custom microbial phosphatase database based on the results of the search described above. We retrieved all UIDs with the annotation of “alkaline phosphatase”, “PhoD-like phosphatase”, or “PhoD-like metallophosphatase” from the NCBI nr database using the NCBI API, followed by conversions from UIDs to NCBI accession numbers (ACCs). We referred to the accession2taxid database and exclusively retained ACCs from the identified bacteria, fungi, and archaea (*i.e.*, unidentified environmental sequences were excluded). These accession numbers were subjected to a blastdbcmd search against the nr database, which retrieves amino acid sequences corresponding to the accession numbers.

The translated sequences were subjected to a blastp search against this custom database with a maximum E-value of 1E-05 and minimum bit score of 100. Taxonomic annotations were obtained using the lowest common ancestor (LCA) algorithm implemented in MEGAN 6.8.12 (26), based on the blastp result. Sequences that were not annotated as *phoD* genes were excluded from downstream analyses. In addition, in order to exclude intracellular phosphatase genes, we performed protein subcellular localization predictions using PSORTb 3.0 (55). Sequences predicted to be “cytoplasmic” or “cytoplasmic membrane” were discarded. The representative nucleotide sequences of all OTUs were subsequently aligned using the MUSCLE algorithm (15) facilitated in MacQIIME ver 1.9.1, followed by the construction of a phylogenetic tree based on the FastTree algorithm (41) in preparation for further statistical analyses described below.

Additionally, in order to compare our results with previously reported *phoD* compositions in forest soil, grassland soil, and arable soil other than Andisols, pyrosequencing data under project IDs ERP012746 and ERP008947 (43) were retrieved from ENA. These two projects were selected because the primers used to amplify *phoD* were the same as the present study. The retrieved sequences were mapped to the above-described OTUs, following quality filtering.

### Phylogenetic analysis of *phoD*

In order to confirm the validity of the taxonomic annotation of *phoD* OTU representative sequences, *phoD* OTU sequences were phylogenetically analyzed. Representative sequences of the 50 most frequently observed OTUs (*i.e.*, OTUs with the highest average relative abundance among 43 samples) were selected. Additionally, four frequently observed OTUs annotated as phylum *Planctomycetes* were selected in order to compare our results with previous findings, among which *Planctomycetes phoD* was frequently observed (43). The 54 selected sequences were aligned by MUSCLE facilitated in MacQIIME ver 1.9.1, followed by the construction of a phylogenetic tree using the FastTree algorithm. The reliability of the phylogenetic tree was evaluated by a bootstrapping test (1,000×iterations) using seqboot implemented in the PHYLIP package (17).

### Statistical analyses

In order to remove the bias caused by differences in sequencing depths, OTU compositions were randomly rarefied to 11,333 and 2,000 reads per sample of the 16S rRNA gene and *phoD*, respectively. Weighted UniFrac distances (33) of 16S rRNA gene compositions and *phoD* compositions were calculated. Based on these beta-diversities, two-dimensional non-metric dimensional scaling (2D-NMDS) scores were calculated. Additionally, Shannon indices were calculated based on OTU compositions in order to estimate gene diversity in each community. MacQIIME ver 1.9.1 was used for these analyses, and logarithm base two was employed to calculate the Shannon index. The *phoD* compositions of the Japanese arable Andisols and those of forests and grasslands were compared by hierarchical clustering using R ver 3.4.0 (42). The influence of land use situations on soil chemical characteristics and enzyme activities was examined using the Wilcoxon rank-sum test. In order to evaluate the relationship between soil chemical properties and microbial communities, a distance-based redundancy analysis (dbRDA) and variation partitioning (VP) were performed based on weighted UniFrac distances. The explanatory variables in dbRDA were soil pH, available phosphorus concentrations, and total carbon concentrations. Total nitrogen concentrations, which strongly correlated with total carbon concentrations (Spearman’s rho=0.951), were excluded from dbRDA in order to avoid multicollinearity. Additionally, the Mantel test (1,000×randomization) was employed to test the significance of the relationships between individual chemical characteristics and microbial community compositions. In the Wilcoxon rank-sum test, dbRDA, VP, and Mantel test, the R package “vegan” (Oksanen, J., F.G. Blanchet, M. Friendly, *et al.* 2017. vegan: Community Ecology Package. R Package. version 2.4-4., https://CRAN.R-project.org/package=vegan.) was used in R ver 3.4.0.

### Accession number

Illumina sequencing data were deposited in the DDBJ/ENA/ GenBank database under BioProject ID PRJDB6544.

## Results

### Soil physiochemical and biochemical characteristics

All 43 soil samples showed acidic to circumneutral pH, ranging between 4.8 and 7.1. The concentration of available phosphorus ranged between 10.8 and 308 mg P kg^−1^ soil, and 31 samples were within the recommended range of available phosphorus concentrations (43.6–436 mg P kg^−1^ dry soil; 38) (Fig. S1). Soil total carbon and nitrogen concentrations showed a strong correlation (*P*=2.2×10^−16^, Spearman’s rho=0.951). The activities of acid phosphatase, alkaline phosphatase, and β–D–glucosidase strongly correlated with each other (Fig. S2). Acid and alkaline phosphatase activities normalized (*i.e.*, divided) by microbial activity (β–D–glucosidase activity) also strongly correlated (*P*=1.84×10^−7^, Spearman’s rho=0.729). In addition, acid phosphatase activities were generally stronger than alkaline phosphatase activities (1.40±0.36 times [mean±SD]), with the exception of KS samples (0.725–0.940-fold). None of the chemical characteristics or enzyme activities were significantly different between planted and unplanted soil (Wilcoxon rank-sum test, *P*>0.05).

### Bacterial community structure, diversity, and relationship with soil chemical properties

**Fig. 2:**
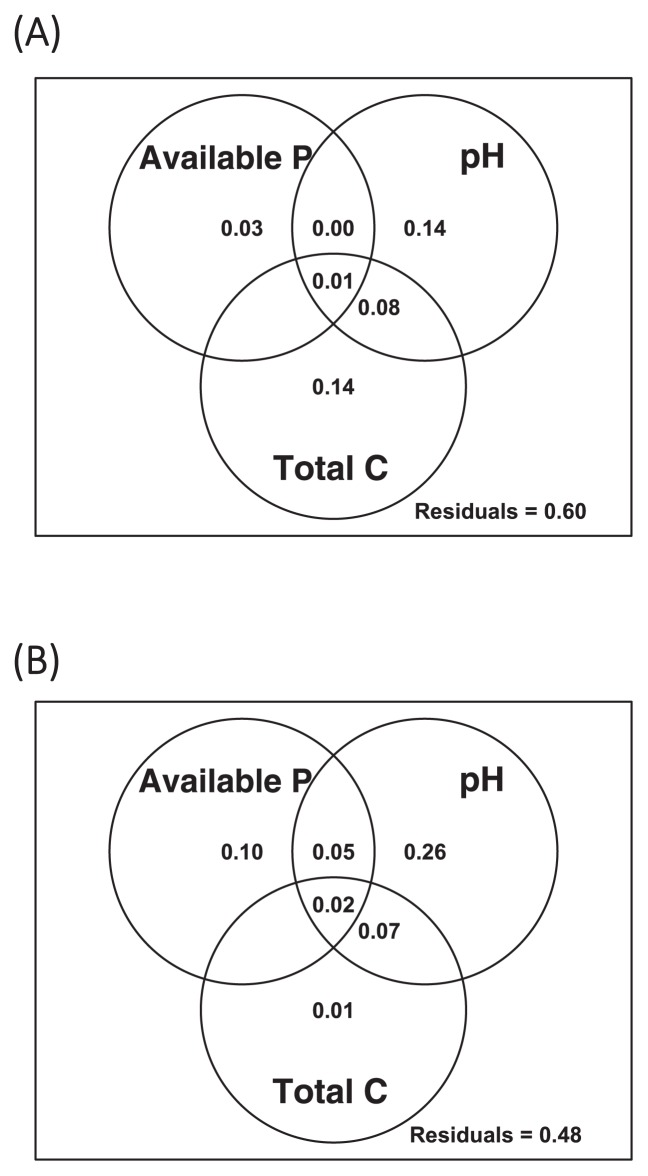
Variation partitioning of (A) the 16S rRNA gene and (B) *phoD* operational taxonomic unit (OTU) compositions. Numerals in the Venn diagram indicate the fraction of variation explained by each soil chemical factor or jointly by two or three factors. Negative values are not shown in the diagram.

A total of 12,918 OTUs were obtained by OTU clustering, 360 of which were annotated as chloroplasts or mitochondria, or unidentified at the domain level. Although the rarefaction curves of the observed OTUs indicated that sequencing was not sufficiently deep to detect all rare-biosphere taxa (Fig. S3A), the major taxonomic groups were all covered (Fig. S3B, C, D, and E). Shannon indices ranged between 8.03 and 9.88, and no clear correlation was observed with soil biochemical/chemical properties (Spearman’s rank correlation test, *P*>0.05). The bacterial community structure at the phylum level was similar between the samples (Fig. S4). The dominant phyla were *Proteobacteria* (24.5–37.0%), *Acidobacteria* (12.1–29.0%), and *Actinobacteria* (7.36–22.5%). The proportions of the phyla *Firmicutes* and *Verrucomicrobia* varied broadly between samples: 1.01–17.1% and 2.81–11.0%, respectively. Mantel tests based on weighted UniFrac distances between communities showed correlations between bacterial community structures and soil pH, available phosphorus concentrations, and soil total carbon and nitrogen concentrations (Table 1). However, variation partitioning and 2D-NMDS plots indicated the smaller contribution of available phosphorus concentrations to the overall prokaryotic community structure than other factors (Fig. 2A and S5).

### Diversity and community composition of phoD-harboring microbes

**Fig. 3:**
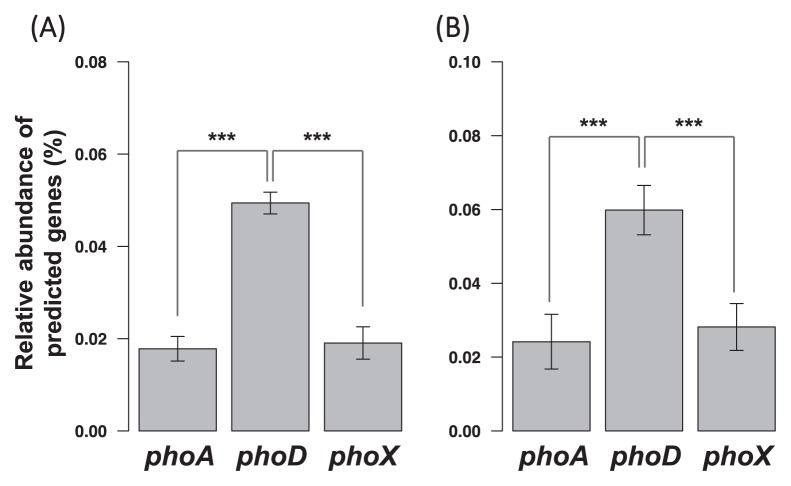
Relative abundance of three clades of alkaline phosphatase genes (mean±SD), predicted by (A) PICRUSt and (B) Piphillin. Both predicted that *phoD* was significantly more abundant than the other two (the Wilcoxon rank-sum test, ***P<2.2E-16).

Phosphomonoesterase gene content was predicted based on 16S rRNA gene compositions. The proportion of *phoD* was estimated to be significantly higher than those of *phoA* and *phoX* (Fig. 3). The composition of *phoD* genes, which were estimated to be more abundant and ubiquitous than the other phosphatase genes, was analyzed by *phoD*-targeted amplicon sequencing. A total of 1,710 OTUs were obtained by OTU clustering, and 1,171 OTUs (which accounted for 70.5–94.1% of the total reads within each sample) were annotated as phosphatase genes in a blastp search against the NCBI nr-database (Table S3). On the other hand, 284 OTUs (accounting for 4.61–28.6% of the total reads within each sample) hit “hypothetical” genes (Table S3), which obscured the results of the blastp search. In order to enable a more precise analysis of *phoD*-harboring communities, an in-house reference database containing only phosphatase genes was constructed. The blastp search against the phosphatase-specific database annotated 1,410 OTUs (accounting for 93.4–99.9% of the total reads within each sample) as phosphatase genes. After the removal of OTUs predicted to be cytoplasmic, the remaining 1,387 OTUs (accounting for 91.0–98.5% of the total reads within each sample) were subjected to downstream analyses.

Overall, the composition of *phoD* correlated with soil pH and available phosphorus concentrations, but not with soil total carbon or nitrogen concentrations (the Mantel test, Table 1). Variation partitioning and 2D-NMDS plots also demonstrated the strong effect of pH and available phosphorus concentrations on the composition of *phoD* (Fig. 2B and S7).

Taxonomic annotations by the blastp search showed that *phoD* of the class *Alphaproteobacteria* accounted for 34.4–89.2% of the total reads. Other frequently observed OTUs were those of the class *Betaproteobacteria* (4.20–21.9%) and the phyla *Cyanobacteria* (0.85–21.8%) and *Actinobacteria* (1.05–10.6%). The composition of *phoD* markedly differed from previously reported *phoD* communities (43, 44) that were investigated using the same primer set as that used in the present study (Fig. S6).

**Fig. 4:**
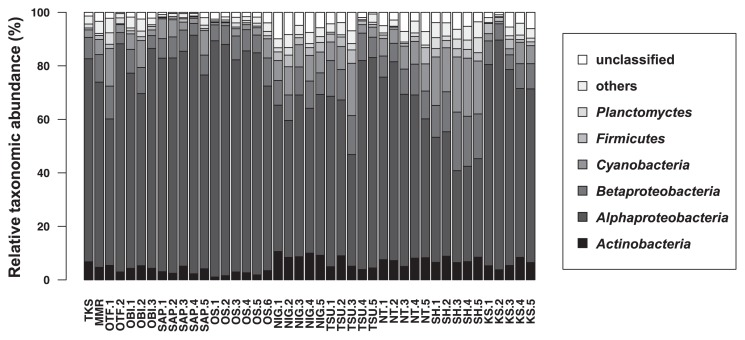
Taxonomic composition (phyla and classes) of *phoD* in 43 soil samples.

**Fig. 5:**
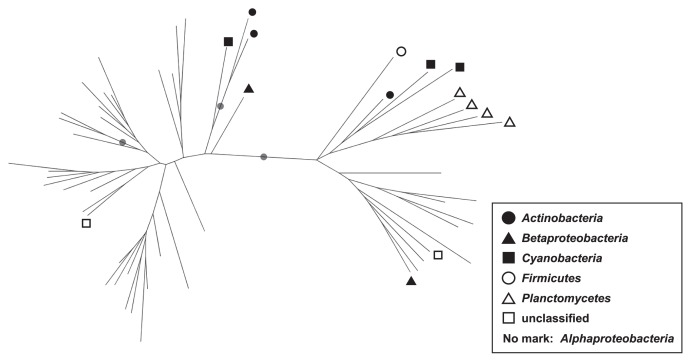
Phylogenetic tree consisting of 54 representative *phoD* operational taxonomic units (OTUs). Filled circles on branches indicate bootstrap value ≥0.95. Leaf nodes without symbols are those of the class *Alphaproteobacteria*.

The taxonomic composition of *phoD* was more divergent between soils than that of the 16S rRNA gene (Fig. 4 and S4). The phylogenetic tree consisting of the representative sequences of 54 OTUs showed with high confidence that alphaproteo-bacterial OTUs were phylogenetically distinct from the others (Fig. 5, bootstrap value=1.00). In contrast, the phylogenies of the OTUs of the phylum *Firmicutes*, phylum *Cyanobacteria*, phylum *Planctomycetes*, and class *Betaproteobacteria* were not distinctive from each other (Fig. 5).

## Discussions

The soil pH in our samples ranged between 4.8 and 7.1 (Fig. S1A), a range that includes the typical optimum pH of acid phosphatase (37). However, this does not suggest that alkaline phosphatase genes play a marginal role in phosphorus cycling in agricultural Andisols. In the present study, phosphomonoesterase activities at pH 11 were 76.8±22.4% (mean±SD) of those at pH 6.5. In contrast, previous studies reported that phosphomonoesterase in acidic soils was approximately five-to ten-fold less active at pH 11 than at pH 6.5 (16, 19, 29). Thus, the relatively strong phosphomonoesterase activities observed at pH 11 in the present study cannot be solely explained by the activity of acid phosphatases. Hence, alkaline phosphatase genes appeared to be expressed in our samples. Furthermore, it has been suggested that alkaline phosphatase is still active in neutral or acidic environments (37). Therefore, we suspect that alkaline phosphatase plays important roles in phosphorus cycling in agricultural Andisols, including acidic types.

Among the three clades of phosphatase genes, *phoA*, *phoD*, and *phoX*, the most abundant was *phoD* (Fig. 3). Although metagenomic predictions have sometimes been pointed out to be inaccurate, this result was consistent between two tools based on different algorithms (27, 30), which strengthens the confidence of this result. Notably, our result supports the approach employed by most previous studies, which targeted the *phoD* gene rather than *phoA* and *phoX*. The widespread use of this approach is partly due to the first PCR primers targeting soil bacterial alkaline phosphatase (46) covering *phoD* exclusively, although seemingly unintentionally (43). More recently, genomic investigations have presumed that *phoD* is predominant in terrestrial ecosystems because *phoD* is more widely distributed than *phoA* and *phoX* among terrestrial microbes (50). Our functional gene predictions supported this presumption in various agricultural Andisols. Therefore, we focused on *phoD* diversity rather than that of *phoA* or *phoX*.

As indicated in Fig. 4, alphaproteobacterial *phoD* was more widely observed than in previous studies conducted on various types of soils. Zimmerman *et al.* (57) reported that extracellular phosphatase genes were widely distributed taxonomically and markedly polyphyletic. This may be due to horizontal gene transfer (HGT), which obscures the relationship between *phoD* phylogeny and 16S rRNA gene phylogeny (or bacterial taxonomy) (24). Therefore, difficulties may be associated with examining taxonomy based on partial *phoD* sequences amplified by PHOD-F733/R1083 primers. However, most *phoD* sequences of the class *Alphaproteobacteria* were distinct from those of other bacteria, such as the phyla *Planctomycetes*, *Firmicutes*, and *Cyanobacteria* (Fig. 5) which were previously reported to be dominant *phoD*-harboring microbes in grasslands (43). Thus, we assume that *phoD*-harboring communities in Japanese Andisols are clearly different from those of grassland, forest, and non-Andisols arable soil. The class *Alphaproteobacteria* is physiologically copiotrophic, favoring nutrient-rich environments (18). Hence, we suspect that the difference in *phoD*-harboring communities may be partly attributable to intensive fertilization practices in the Japanese Andisols (48); however, the direct validation of this hypothesis is beyond our analyses.

Statistical analyses showed that the 16S rRNA gene composition was mainly influenced by soil pH, soil carbon concentrations, and soil nitrogen concentrations, whereas *phoD*-harboring communities were primarily affected by soil pH and available phosphorus concentrations (Table 1, Fig. 2, S5 and S7). Regarding pH, these results are consistent with previous findings, which argued that bacterial community compositions and *phoD* gene compositions in grasslands and forests were mainly influenced by soil pH (31, 43). Our analyses expanded these insights to arable Andisols, which were impacted by anthropogenic activities. In contrast, a previous inter-continental scale study did not find a correlation between *phoD*-harboring community compositions and soil pH (44). This finding, together with the present results, indicates that the distribution of the phosphorus-mineralizing microbial community is dependent on the spatial scale.

In accordance with our results, soil phosphorus content has also been reported to affect the *phoD*-harboring community (44). It is important to note that we cannot directly compare our results with previous findings because the assessment of phosphorus content is dependent on the extraction method and soil physicochemical properties, such as aluminum oxide content (1) and soil particle size (25), which differ among studies. Additionally, the primers employed in previous studies were biased to the order *Rhizobiales* (43, 50). Thus, the influence of soil phosphorus content on the phosphorus-mineralizing community remains hypothetical.

Contrary to our results, some findings argued that soil carbon or nitrogen content correlated with the composition of *phoD* (28, 44). This difference may be attributed to Andisols generally being rich in carbon and nitrogen (48). The soil total nitrogen content reported by Ragot *et al.* (44) was 1.7±1.0 g N kg^−1^ dry soil (mean±SD), which is clearly lower than in our samples (Fig. S1D). Due to the richness of carbon and nitrogen in Andisols, their availability or scarcity may have had less of an impact on *phoD*-harboring community assembly.

In conclusion, we characterized the microbial mineralization of organic phosphorus in arable Andisols. Alkaline phosphatase was estimated to play important roles in arable Andisols, including acidic ones, based on soil biochemical analyses. Functional gene predictions suggested that *phoD* was more abundant than the other microbial alkaline phosphorus genes in arable Andisols. Amplicon sequencing targeting *phoD* showed that the phosphorus-mineralizing community in arable Andisols is characterized by the dominance of *Alphaproteobacteria*, which suggests the influence of nutrient-rich conditions on the soil microbial community. The overall *phoD* composition was hypothetically assessed by soil pH and soil phosphorus content, rather than by soil carbon and nitrogen contents.

## Supplementary Material



## Figures and Tables

**Table 1:** Mantel correlations between (A) prokaryotic and (B) *phoD*-harboring community compositions and soil chemical factors.

	(A) Prokaryotic community composition	(B) *phoD*-harboring community composition
pH	0.301 ***	0.605 ***
Available phosphorus	0.106 *	0.365 ***
Total carbon	0.288 ***	0.049
Total nitrogen	0.228 ***	0.029

*** *P*<0.001,

** *P*<0.01,

* *P*<0.05
