# Supplementary material for: Phosphorus-mineralizing Communities Reflect Nutrient-Rich Characteristics in Japanese Arable Andisols
Source: Microbes Environ. 2018 Sep 29;33(3):282–9. doi: 10.1264/jsme2.ME18043 (PMC6167123; doi:10.1264/jsme2.ME18043)
Supplement: Supplementary file 1 [file 33_282_s1.pdf]

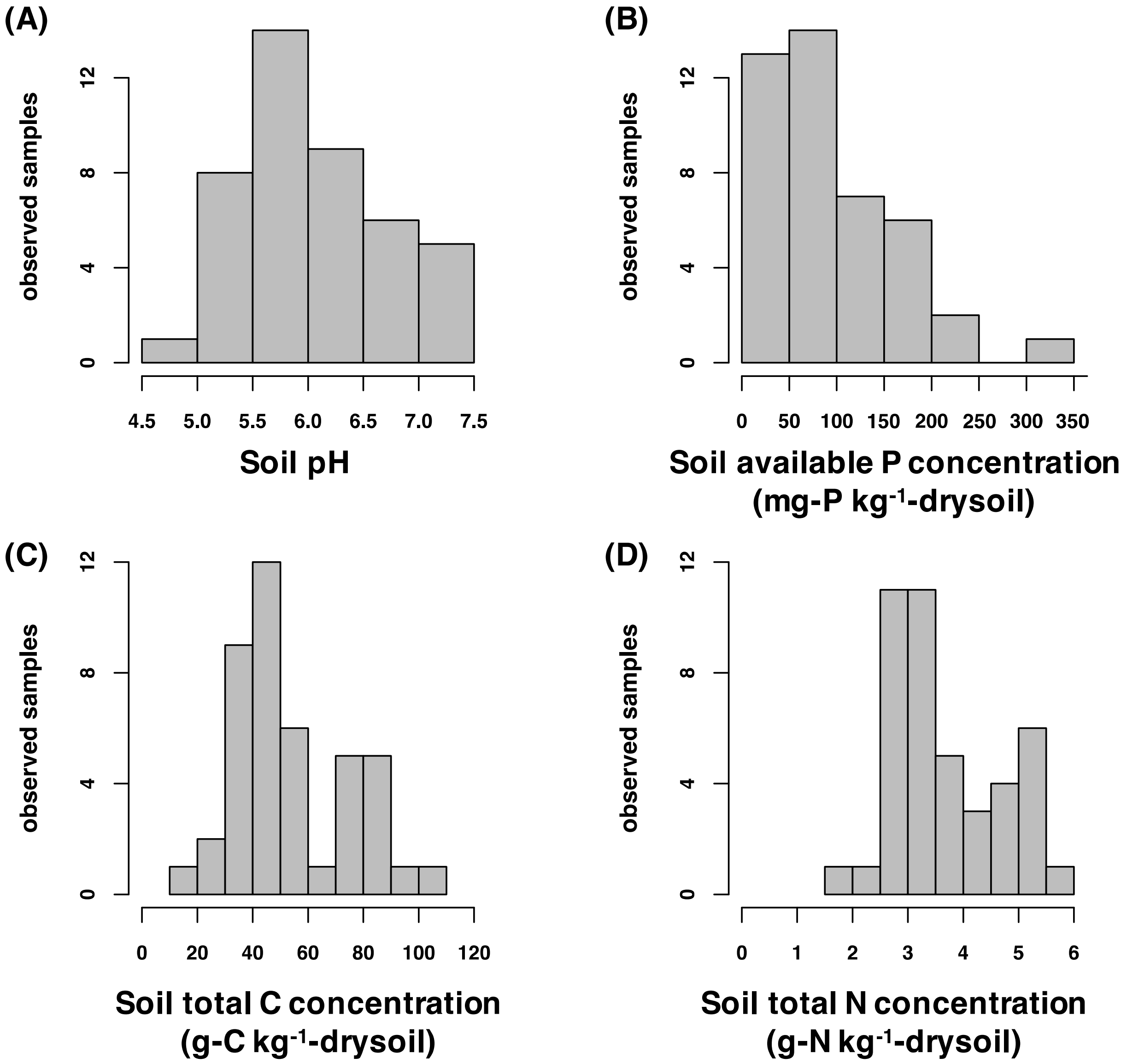

Supplementary Figure 1. Overall trend of soil chemical characteristics: (A) soil pH, (B) available phosphorus concentration, (C) total carbon concentration, and (D) total nitrogen concentration.

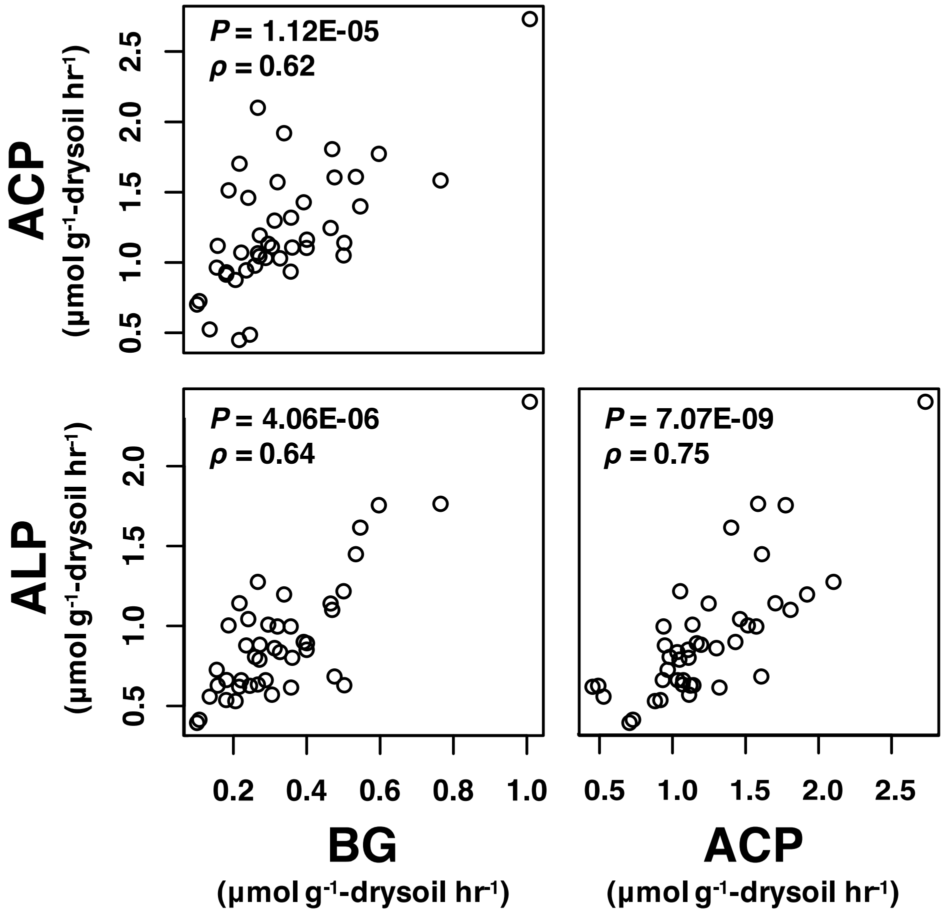

Supplementary Figure 2. Correlations between soil enzyme activities. Each circular plot corresponds one soil sample. Spearman's rho and *p*-value (based on Spearman's rho) of each correlation are also presented.

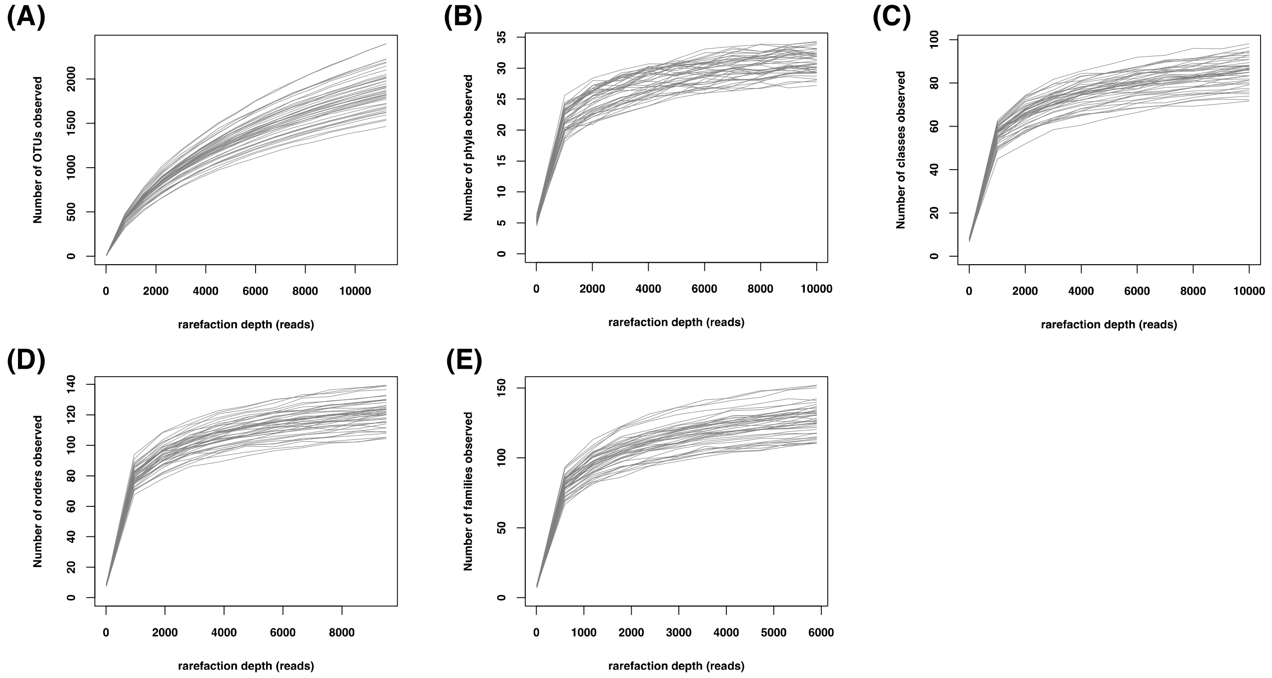

Supplementary Figure 3. Rarefaction curve of 16S amplicons, indicating the number of observed (A) operational taxonomic units (OTUs), (B) phyla, (C) classes, (D) orders, and (E) families.

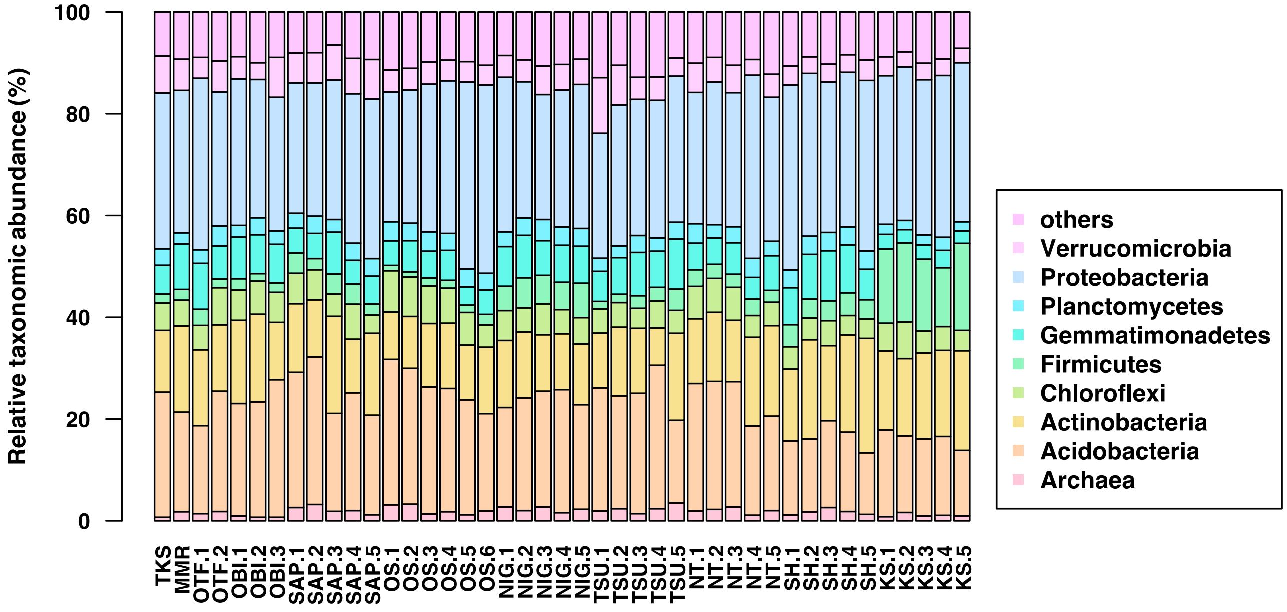

Supplementary Figure 4. Taxonomic composition of 16S rRNA genes in 43 soil samples. Organellar and unidentified genes at domain level were excluded. Bacterial taxa with average relative abundance of ≤ 5% are presented as “others.”

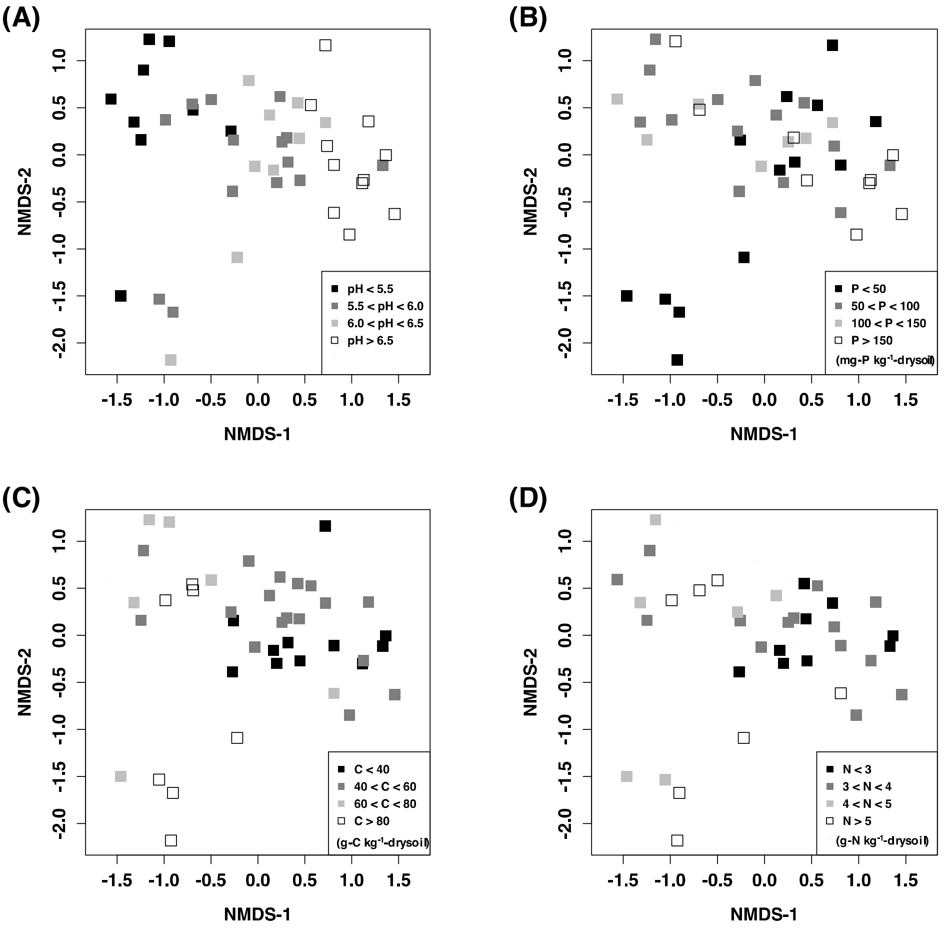

Supplementary Figure 5. Relationship between prokaryotic 16S rRNA composition and chemical properties: (A) soil pH, (B) available phosphorus concentration, (C) total carbon concentration, and (D) total nitrogen concentration visualized using two-dimensional (2D)-non-metric multidimensional scaling (NMDS) plots based on weighted UniFrac distances (2D-stress=0.13).

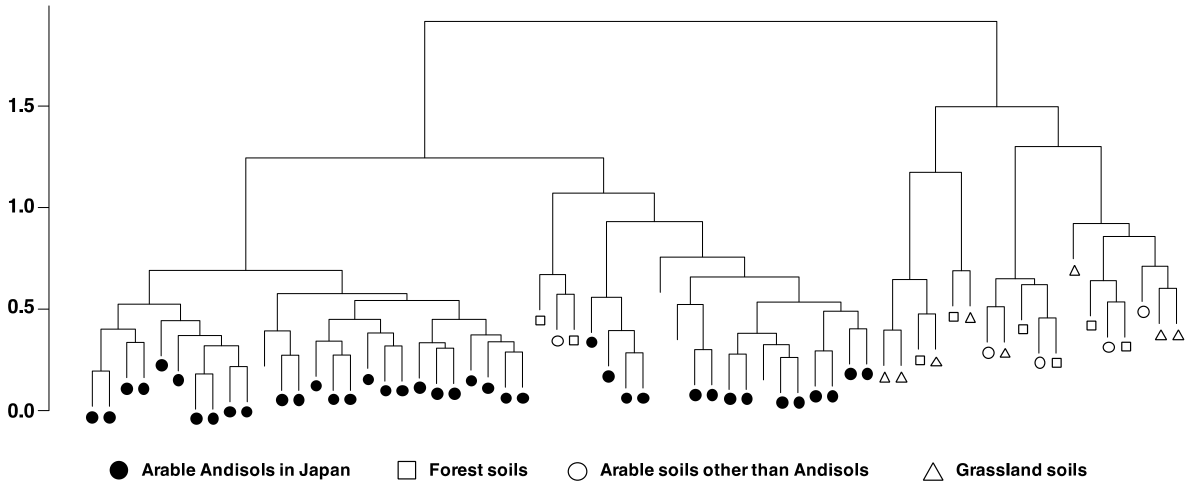

Supplementary Figure 6. Comparison of *phoD*-harboring community compositions between arable Andisols in Japan (our samples), grassland soils (43), arable soils other than Andisols (44) and forest soils (44), illustrated by hierarchy clustering dendrogram based on weighted UniFrac distances.

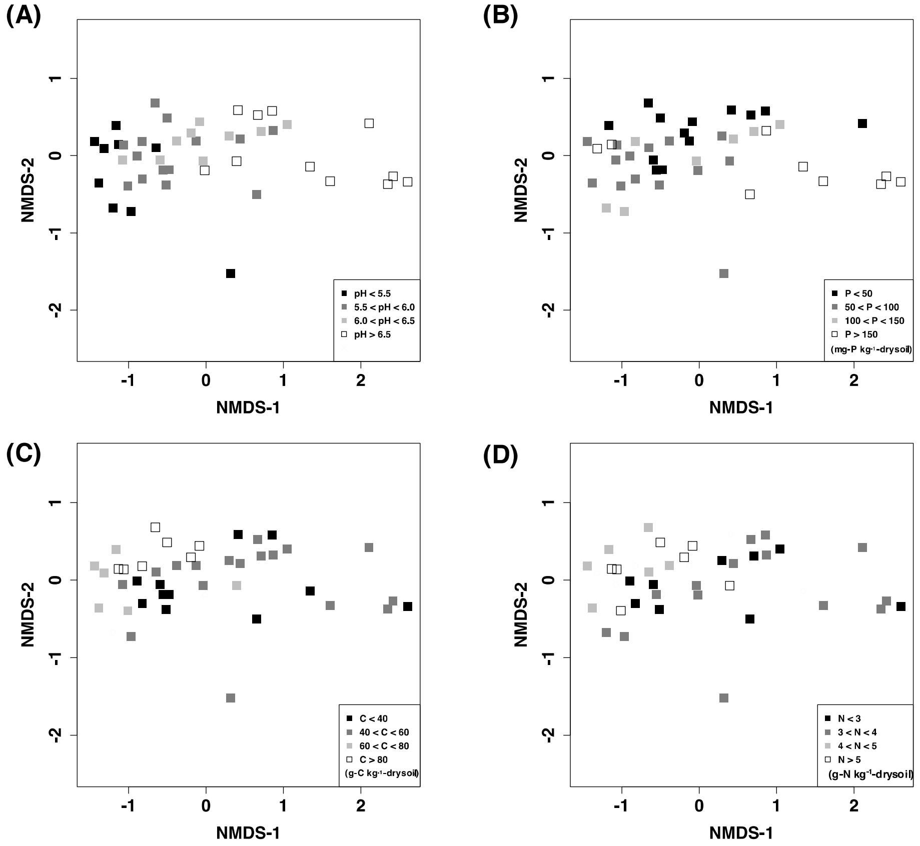

Supplementary Figure 7. Relationship between *phoD* composition and chemical properties: (A) soil pH, (B) available phosphorus concentration, (C) total carbon concentration, and (D) total nitrogen concentration visualized using 2D-NMDS plots based on weighted UniFrac distances (2D-stress=0.09).

Supplementary Table 1. Detailed information in soil samples. Descriptions in land usage follow the information provided by the sampling institutions.

Sample name	Location (Latitude, Longitude)	Land usage in 2016	Institution associated with sampling
TKS	Tokachishimizu (N 43.0, E 142.9)	Chinese cabbages; fertilization not recorded	Tokachi Nokyoren Agricultural Research Institute
MMR	Memuro (N 42.9, E 143.0)	Cabbages; fertilization not recorded	
OTF-1	Otofuke (N 43.0, E 143.3)	Cabbages; fertilization not recorded	
OTF-2		Lettuces; fertilization not recorded	
OBI-1	Obihiro (N 42.8, E 143.1)	Potatoes; fertilization not recorded	
OBI-2		Soy beans; fertilization not recorded	
OBI-3		Kidney beans; fertilization not recorded	
SAP-1	Sapporo (N 43.0, E 141.4)	No crops planted; fertilizer amended	National Agriculture and Food Research Organization
SAP-2		Soy beans; fertilizer amended	
SAP-3		Soy beans; fertilizer amended	
SAP-4		Onions; fertilizer amended	
SAP-5		Soy beans; fertilization not recorded	
OS-1	Osaki (N 38.7, E 140.8)	Green onions; no fertilizer	Tohoku University
OS-2		Green onions; fertilizer amended	
OS-3		Green onions; no fertilizer	
OS-4		Green onions; fertilizer amended	
OS-5		Maize; fertilization not recorded	
OS-6		Potatoes; fertilization not recorded	
NIG-1	Nagaoka (N 37.4, E 138.9	Maize; no nitrogen fertilizer	Niigata Agricultural Research Institute
NIG-2		Maize; fertilizer amended	
NIG-3		No crops planted; fertilization not recorded	
NIG-4		Barley; fertilizer amended	
NIG-5		Soybeans; fertilizer amended	
TSU-1	Tsukuba (N 36.0, E 140.1)	Crops not recorded; no fertilizer	National Agriculture and Food Research Organization
TSU-2		Crops not recorded; fertilizer amended	
TSU-3		No crops planted; fertilization not recorded	
TSU-4		No crops planted; fertilization not recorded	
TSU-5		No crops planted; fertilization not recorded	
NT-1	Nishi-tokyo (N 35.7, E 139.5)	Crops planted; no fertilizer	the University of Tokyo
NT-2		Crops planted; fertilizer amended	
NT-3		Crops planted; fertilizer amended	
NT-4		Crops planted; fertilizer amended	
NT-5		No crops planted; no fertilizer	
SH-1	Shiojiri (N 36.1, E 137.9)	Chinese cabbages; fertilizer amended	Nagano Vegetable and Ornamental Crops Experiment Station
SH-2		Japanese radish; fertilizer amended	
SH-3		Chinese cabbages; fertilizer amended	
SH-4		Japanese radish; fertilizer amended	
SH-5		Crops not recorded; fertilization not recorded	
KS-1	Koshi (N 32.9, E 130.7)	No crops planted; fertilization not recorded	National Agriculture and Food Research Organization
KS-2		Maize; fertilizer amended	
KS-3		Sweet potatoes; fertilization not recorded	
KS-4		Maize; fertilization not recorded	
KS-5		Maize; fertilizer amended	

Supplementary Table 2. PCR conditions tageting (A) 16S rRNA gene V4 region, (B) *phoD* gene.

(A)

ExTaq Buffer	5.0 μL
dNTP mixture(2.5mM each)	4.0 μL
515F primer (10μM)	1.0 μL
806R primer (10μM)	1.0 μL
ExTaq HS (5U/μL)	0.2 μL
DNA template	1.0 μL
ultrapure water	fill up
total volume	50 μL

94°C	3 min.	× 1
94°C	30 sec.	
50°C	45 sec.	× 22
72°C	90 sec.	
72°C	5 min.	× 1
4°C	∞	

(B)

KOD-plus-Neo Buffer	5.0 μL
dNTP mixture(2.0mM each)	5.0 μL
$MgSO_4$ solution	1.6 μL
PHOD-F733 primer (10μM)	1.5 μL
PHOD-R1083 primer (10μM)	1.5 μL
KOD-plus-Neo (1U/μL)	1.0 μL
DNA template	1.0 μL
ultrapure water	fill up
total volume	50 μL

94°C	2 min.	× 1
98°C	10 sec.	
58°C	30 sec.	× 30
68°C	20 sec.	
4°C	∞	× 1

Supplementary Table 3. Frequently-observed annotations of *phoD* operational taxonomic units (OTUs) representative sequences, when searched against NCBI nr database.

annotation	number of OTUs
alkaline phosphatase	515
alkaline phosphatase, partial	364
hypothetical protein	177
PhoD-like metallophosphatase, partial	162
alkaline phosphatase D	54
hypothetical protein BGO98_46540	21
phosphodiesterase/alkaline phosphatase D-like protein, partial	15
secreted alkaline phosphatase	15
phosphodiesterase/alkaline phosphatase D	13
Twin-arginine translocation pathway signal	13
MULTISPECIES: alkaline phosphatase	10
hypothetical protein AUH43_09415	8
PhoD-like phosphatase, partial	7
Alkaline phosphatase D-related protein	6
hypothetical protein, partial	6
alkaline phosphatase D-related protein	5
Alkaline phosphatase	4
Alkaline phosphatase D precursor	4
DNA-binding response regulator	3
hypothetical protein A2W68_08855	3
hypothetical protein A3I02_13850	3
hypothetical protein AUH33_01540	3
Phosphodiesterase/alkaline phosphatase D	3
Alkaline phosphatase D	2
amidohydrolase	2
asparagine synthase (glutamine-hydrolyzing)	2
hypothetical protein A2W08_13305	2
hypothetical protein A3G81_16335	2
hypothetical protein A4E20_17355	2
hypothetical protein AUG87_05210	2
hypothetical protein AUG95_01505	2
peptide ABC transporter substrate-binding protein	2
phoD-like phosphatase family protein	2
Phosphodiesterase/alkaline phosphatase D (fragment)	2
phosphodiesterase/alkaline phosphatase D, alkaline phosphatase D	2
Uncharacterised protein	2
1-deoxy-D-xylulose 5-phosphate reductoisomerase	1
2-polyprenyl-6-methoxyphenol hydroxylase	1
5-carboxymethyl-2-hydroxymuconate isomerase	1
ABC transporter ATP-binding protein	1
ACP synthase	1
acyl-CoA dehydrogenase	1
adenine deaminase	1
alkaline phosphatase D, partial	1
amidase	1
amino acid permease, partial	1
ATP-dependent protease ATP-binding subunit ClpX	1

ATPase	1
cell division protein FtsH	1
chromate resistance protein	1
conjugal transfer protein TraG	1
diguanylate cyclase (GGDEF) domain-containing protein	1
enoyl-CoA hydratase/isomerase family protein	1
esterase	1
excinuclease ABC subunit A	1
flagellar motor switch protein FliG	1
fumarylacetoacetate hydrolase	1
glucosamine-6-phosphate isomerase	1
glucosidase	1
glutamate dehydrogenase	1
glycoside hydrolase family 31	1
glycosyl transferase	1
GNAT family N-acetyltransferase	1
hydrogenase expression/formation protein HypE	1
hydrolase TatD	1
hypothetical protein A176_005183	1
hypothetical protein A2283_20320	1
hypothetical protein A2V88_14935	1
hypothetical protein A2Z17_03980	1
hypothetical protein A2Z37_05060	1
hypothetical protein A3194_17385	1
hypothetical protein A3F84_11140	1
hypothetical protein A3H34_07570	1
hypothetical protein A3H96_23325	1
hypothetical protein A3I01_19835	1
hypothetical protein ABS52_05670	1
hypothetical protein ACD_75C01299G0001	1
hypothetical protein AGR7B_Cc10313	1
hypothetical protein AKJ09_00690	1
hypothetical protein AMJ58_10435	1
hypothetical protein AMJ79_11660	1
hypothetical protein AUG08_01360	1
hypothetical protein AUG48_04640	1
hypothetical protein AUG51_18835	1
hypothetical protein AUG88_05450	1
hypothetical protein AUH07_07145	1
hypothetical protein AUH30_05700	1
hypothetical protein AUH32_07100	1
hypothetical protein AUH43_02410	1
hypothetical protein AUH74_04195	1
hypothetical protein AUI36_08655	1
hypothetical protein AUI36_35965	1
hypothetical protein AUI48_08225	1
hypothetical protein AUI54_01395	1
hypothetical protein AUJ04_08645	1
hypothetical protein AUJ06_02580	1
hypothetical protein AX769_02925	1
hypothetical protein Barb7_03162	1
hypothetical protein BB934_34760 (plasmid)	1
hypothetical protein BGN86_02985	1
hypothetical protein BGN99_05650	1
hypothetical protein BGO51_21195	1

hypothetical protein BN1723_009833, partial	1
hypothetical protein BN961_01903	1
hypothetical protein BURPS1710b_2316	1
hypothetical protein COLAER_01042	1
hypothetical protein GY15_12955	1
hypothetical protein MPTA5024_12635	1
hypothetical protein NM3141_2205	1
hypothetical protein OG2516_09523	1
hypothetical protein PAERUG_P53_London_9_VIM_2_02_13_03122	1
hypothetical protein SAMN02982931_00975	1
hypothetical protein SAMN04487897_11321	1
hypothetical protein SAMN04515617_111152	1
hypothetical protein SE17_05640, partial	1
hypothetical protein SP67_18705, partial	1
hypothetical protein XU10_C0021G0002	1
L-lactate dehydrogenase	1
mannose-1-phosphate guanylyltransferase	1
metal-binding protein	1
MFS transporter	1
Microsomal dipeptidase	1
MULTISPECIES: hypothetical protein	1
N-acetyltransferase	1
NAD-dependent succinate-semialdehyde dehydrogenase	1
penicillin acylase family protein	1
phosphotransferase family protein	1
polysaccharide deacetylase	1
PREDICTED: alkaline phosphatase D-like	1
pyruvate dehydrogenase (acetyl-transferring), homodimeric type	1
radical SAM protein	1
Ribosomal protein S18 acetylase RimI	1
RNA polymerase sigma24 factor	1
saccharopine dehydrogenase	1
serine hydrolase	1
signal transduction histidine kinase	1
single-stranded DNA-binding protein	1
Tat (twin-arginine translocation) pathway signal sequence domain protein	1
thioredoxin	1
uncharacterized protein STAUR_7361	1
UTP--glucose-1-phosphate uridylyltransferase	1
WD-40 repeat-containing protein	1
